# Effects of “fixation-fusion” sequence of lumbar surgery on surgical outcomes for patients with lumbar spinal stenosis: study protocol for a multicenter randomized controlled trial

**DOI:** 10.1186/s12891-023-07052-y

**Published:** 2023-12-01

**Authors:** Weicheng Pan, Jialin Jiang, Weihang Zhang, Zijian Mei, Kaiqiang Sun, Bing Zheng, Yake Meng, Yushu Bai, Zhimin He, Jiangang Shi, Yongfei Guo

**Affiliations:** 1 Department of Orthopedic Surgery, Spine Center, Changzheng Hospital, Naval Medical University, No. 415 Fengyang Road, Shanghai, 200003 People’s Republic of China; 2 Departmentof Orthopedic Surgery, Spine Center, Changhai Hospital, Naval Medical University, No.168 Changhai Road, Shanghai, 200438 People’s Republic of China; 3 Department of Orthopedic Surgery, Spine Center, Shanghai Tenth People’s Hospital, Tongji University, No. 301 Yanchang Middle Road, Shanghai, 200072 People’s Republic of China

**Keywords:** Modified LIF sequence, Lumbar spinal stenosis, Neurological symptom, Randomized controlled trial

## Abstract

**Background:**

New-onset neurological symptoms such as numbness and pain in lower extremities might appear immediately after conventional lumbar interbody fusion (LIF) surgery performed in patients with lumbar spinal stenosis.

**Methods and analysis:**

This is a multicenter, randomized, open-label, parallel-group, active-controlled trial investigating the clinical outcomes of modified LIF sequence versus conventional LIF sequence in treating patients with lumbar spinal stenosis. A total of 254 eligible patients will be enrolled and randomized in a 1:1 ratio to either modified LIF sequence or conventional LIF sequence group. The primary outcome measure is the perioperative incidence of new-onset lower extremity neurological symptoms, including new adverse events of pain, numbness, and foot drop of any severity. Important secondary endpoints include visual analogue scale (VAS) pain score and lumbar Japanese Orthopaedic Association (JOA) recovery rate. Other safety endpoints will also be evaluated. The safety set used for safety data analysis by the actual surgical treatment received and the full analysis set for baseline and efficacy data analyses according to the intent-to-treat principle will be established as the two analysis populations in the study.

**Conclusion:**

This study is designed to investigate the clinical outcomes of modified LIF sequences in patients with lumbar spinal stenosis. It aims to provide clinical evidence that the modified “fixation-fusion” sequence of LIF surgery is effective in treating lumbar spinal stenosis.

**Trial registration:**

http://www.chictr.org.cn/index.aspx ID: ChiCTR2100048507.

## Introduction

Lumbar spinal stenosis (LSS) may lead to severe pain in low back or leg and also neurogenic claudication [1]. In clinical practice, the lumbar interbody fusion (LIF) technique has been widely used in the treatment of LSS patients to restore the height of the intervertebral disc and foramen and also the sagittal volume of the lumbar spinal canal [2].

In recent years, however, the postoperative neurological symptoms after LIF surgery, characterized by new-onset postoperative pain or numbness, dysesthesia, and muscle weakness have aroused widespread concern among clinicians [3–7]. Although these symptoms are usually transient and would be significantly relieved by themselves, it has a great impact on the quality of life of patients at that time interval. Some researchers have hypothesized about what causes these symptoms. Postigo et al [8]reported an increased risk of numbness, pain, and even foot drop after over-distraction on LIF surgery. A study by Matsui et al [9]showed that during the process of nerve root being pulled, the nerve root usually has ischemia, and hence the traction pressure and duration may be the potential risk factors for nerve root injury. Fu et al [10] found that when the height of the intervertebral space was increased to 140% of the original height, the nerve root tension increased the risk of injury significantly.

These findings coincide with our clinical experience in recent prospective research [11]. In this prospective observational clinical trial, we observed that fixation followed by fusion implantation resulted in a more physiologic reconstruction of the intervertebral space height, rather than excessive distraction resulting in excessive tension of the walking nerve roots, and reduced the incidence of postoperative neurological symptoms. It is essential to acknowledge that previous studies have concluded that additional fusion in conjunction with decompressive surgery may not yield discernible benefits, furthermore, they have noted potential drawbacks such as increased hospitalization time and blood loss [12–14]. The pressing challenge, therefore, lies in the quest to enhance the outcomes of LIF surgery. By doing so, we can bridge the gap between fusion and non-fusion surgery, catering to the unique needs of patients who may still require fusion surgery, and offering a more tailored approach that optimizes the surgical outcomes.

As a result, we modified the LIF surgery technique further, to relieve the nerve root axial traction caused by the excessive distraction of intervertebral space to a greater extent [11, 15–18]. Herein we present the study rationale and methodology for this randomized, controlled trial to investigate the clinical outcomes of lumbar surgery sequence of fixation-fusion in LSS patients. It aims to provide more clinical evidence that the modified sequence of the LIF surgery is effective in treating LSS.

## Methods/design

### Study design

**Fig. 1:**
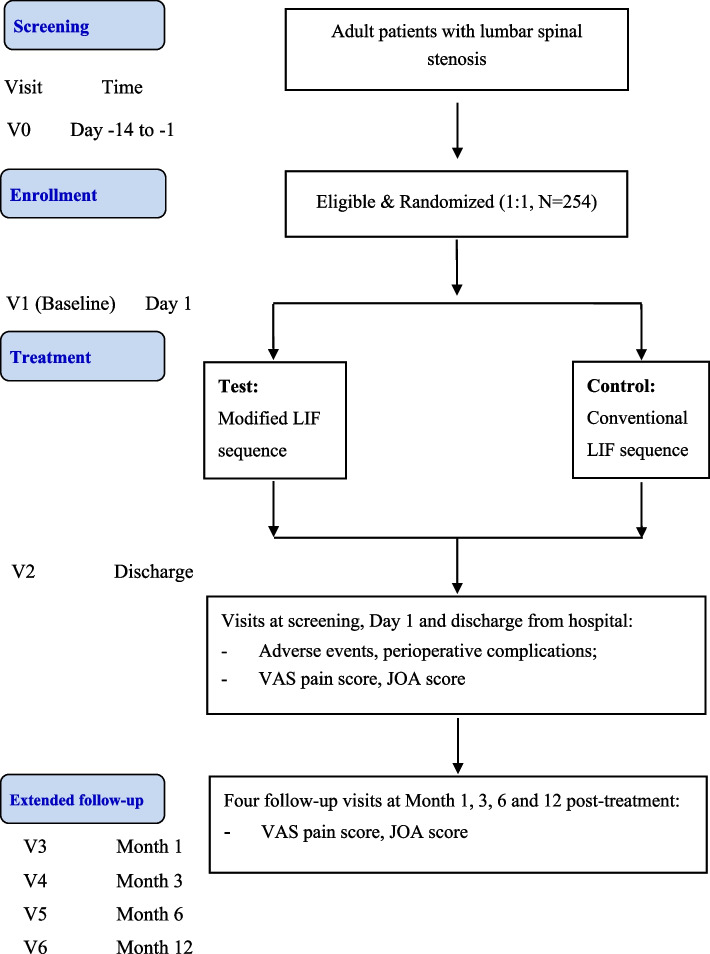
Study flowchart

The study (http://www.chictr.org.cn/index.aspx ID: ChiCTR2100048507) is a multicenter, randomized, open-label, parallel-group, active-controlled trial that will investigate the clinical outcomes of modified LIF sequence versus conventional LIF sequence in treating patients with lumbar spinal stenosis. It will be conducted following the international Consolidated Standards of Reporting Trials (CONSORT) statement (http://www.consort-statement.org/). Approximately three clinical centers will participate in this study. A brief flow chart of this study is provided in Fig. 1.

### Study patients

A total of 254 eligible patients will be enrolled and randomized after screening at the study sites.

The following are the inclusion criteria:

- Age > 18 and < 80 years;
- Non-pregnant and lactating women;
- The patient is suffering from lumbar spinal stenosis (refers to the clinical syndrome of the nerve root and cauda equina compression caused by factors such as facet joint hyperplasia and stimulation, which is manifested as low back pain, lower extremity pain, lower extremity numbness, lower extremity weakness, intermittent claudication, and even urine and stool dysfunction);
- Lumbar spine imaging examination (X-ray film, computed tomography, and magnetic resonance imaging) to diagnose lumbar spinal stenosis, or hyperplasia of facet joint leading to nerve tissue (spinal cord or nerve root) compression, or nerve tissue compression caused by the stenosis of nerve root outlet. On the basis of imaging examination, patients with the Modified Schizas Classification Grade B, C, and D can be enrolled;
- With significant clinical symptoms of lumbar spinal stenosis, including low back pain, lower limb numbness and pain, and intermittent claudication; On physical examination, the patient had limited lumbar extension, positive or negative straight leg elevation test, and abnormal knee and tendon reflexes;
- More than 6 months of conservative treatment, no significant improvement of symptoms, seriously affect the quality of life of patients;

Patients with a strong desire to undergo surgery and a full understanding of the differences between the two surgical procedures and their complications signed an informed consent form to voluntarily participate in the clinical trial.

The exclusion criteria are as follows:

- Patients with cardiovascular, liver, kidney and hematopoietic systems and other serious primary diseases, mental and malignant diseases such as tumors;
- Combined with congenital lumbar malformation, past infection history, tumor history, and trauma history, leading to significant abnormal changes in the shape of the lumbar spine;
- The existence of compression diseases in other parts of the spine other than the lumbar spine, such as cervical disc degeneration, cervical hyperextension, cervical posterior longitudinal ligament ossification, thoracic posterior longitudinal ligament ossification, thoracic ligament yellow ossification, etc.;

There are other diseases such as piriformis syndrome, sciatic nerve injury, polio, and Guillan-Barre syndrome, which affect neurological function and thus interfere with postoperative efficacy.

### Recruitment and randomization process

Before enrollment, there will be one pretreatment screening visit at the study site office, during which each subject will be assigned a unique identification number.

Once considered eligible for entry, the patients with LSS will be randomly assigned to one of two study treatment groups, e.g., either modified LIF sequence or conventional LIF sequence in a 1:1 ratio. A stratified block randomization with randomly varying block size will be used, stratified by surgical segments of lumbar intervertebral disc (single versus multiple segments), modified Schizas classification of LSS (level B versus C/D). Random assignment is generated by an independent statistician and implemented via central randomization mobile phone APP (Shanghai KNOWLANDS MedPharm Consulting Co., Ltd.). In order to avoid potential selection bias, the randomization sequence is concealed from both clinical staff and patients until assignment. With these, neither site investigators nor study participants can influence the study patients’ assigned treatment group.

### Description of the interventions

**Fig. 2:**
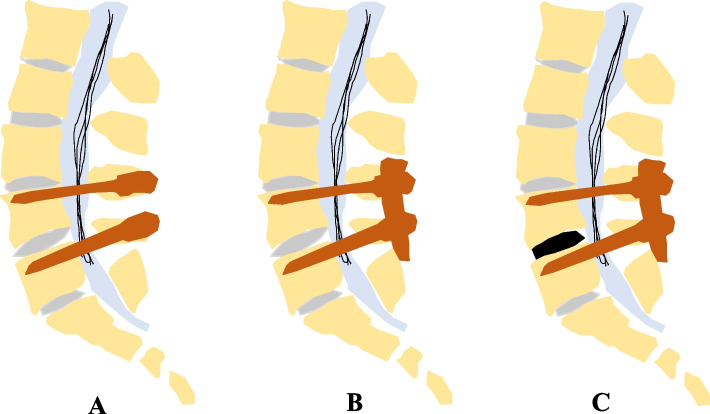
Representative intraoperative images of modified LIF sequence surgery **A**: The insertion of the pedicle screws; **B**: The installation of the rods prior to the placement of cages; **C**: Direct decompression and insertion of the cage after installation of the rods

The enrolled subjects will be randomized to undergo LIF surgery with a modified sequence or a conventional sequence. Electromyography serves as a crucial supplementary assessment and prognostic indicator, to minimize the potential bias of the trial data, each participant will undergo electromyography prior to surgery, mitigating the impact of individual patient factors on the prognosis and ensuring the accuracy of the test results. All subjects would be operated on by senior spine surgeons at each site who have at least five years of spine surgical experience and performed more than 100 cases of LIF surgery annually. All subjects will take the same surgical devices. PEEK was uniformly used as the cage material due to practical limitations. Due to practical limitations, the expandable cage was not used in this clinical trial. Therefore, only static cage is used uniformly for all patients. Representative intraoperative images of modified LIF sequence surgery is provided in Fig. 2.

All the consumables used in the operation are from the same manufacturer, and the operations will be carried out in strict accordance with a unified standard. The only difference is that patients in the modified LIF sequence group are to be implanted with the fusion cage before with the titanium rod, as opposed to those in the conventional LIF sequence group which are in the opposite order. A detailed description of the LIF surgery technique can be found in previous studies [11, 19].

### Study visits

Seven study visits per subject will be scheduled in the study as follows: pretreatment visit (Day − 14 to Day 0), treatment visit (Day 1), discharge visit (on the day of discharge from hospital), follow-up visit month 1 (Month 1 post-treatment), follow-up visit month 3 (Month 3 post-treatment), follow-up visit month 6 (Month 6 post-treatment) and follow-up visit month 12 (Month 12 post-treatment). These visits will be made at the patient ward before discharge or study site office after discharge. At scheduled visits, data relating to demography, operation duration, estimated blood loss during operation period, length in days of the stay at hospital, and perioperative complications, visual analogue scale (VAS) pain score and lumbar Japanese Orthopaedic Association (JOA) score, concomitant medication, new-onset adverse events, etc. will be collected. See Fig. 1 for more details.

In case severe adverse events occur, the subjects can decide to drop out any time during the study.

### Outcome measures

#### Primary outcome

The primary outcome endpoint is the new-onset lower extremity neurological symptoms, including new adverse events of pain, numbness, and foot drop at different severity, which occur on the day of surgery performed until prior to discharge from hospital. In order to exclude the influence of nerve root edema, these new-onset symptoms to be included for analysis should not be significantly relieved three days after conservative treatment of nerve dehydration.

#### Secondary outcomes

The efficacy endpoints in this study mainly included VAS pain score, and lumbar JOA recovery rate pre and post LIF surgery. The VAS pain score is an ordinal scale of 0 to 10 points, with 0 indicating no pain, a higher value indicating more severe pain, and 10 indicating the most severe one. The lumbar JOA recovery rate is derived from lumbar JOA score per subject visit. Lumbar JOA score involves four aspects: subjective symptoms (a range of 0 to 9 points), clinical signs (a range of 0 to 6 points), daily life activities (a range of 0 to 14 points), and bladder function (a range of -6 to 0 points). The total JOA score ranged from − 6 (worst) to 29 points (normal), with a lower score indicating more significant dysfunction. The recovery rate of lumbar JOA is calculated by Hirabayashi’s method [20]as follows:

$$\mathrm{JOA}\;\mathrm{recovery}\;\mathrm{rate}=\frac{\mathrm{Post}\;-\;\mathrm{Preoperative}\;\mathrm{lumbar}\;\mathrm{JOA}\;\mathrm{score}}{29\;-\;\mathrm{Preoperative}\;\mathrm{lumbar}\;\mathrm{JOA}\;\mathrm{score}}\times\;100\%$$

#### Safety outcomes

The safety outcome endpoints also include other adverse events (AEs), surgery complications and laboratory tests as appropriate. The AEs profile of both treatments will be evaluated by examining the incidence of AEs according to the National Cancer Institute Common Terminology Criteria for Adverse Events (NCI CTCAE, Version 5.0, https://ctep.cancer.gov/protocoldevelopment/electronic_applications/docs/CTCAE_v5_Quick_Reference_8.5x11.pdf ).

### Sample size calculation

We used SAS^®^software, V.9.4 (SAS Institute, North Carolina, USA) to estimate sample size. According to the principle of outcome superiority design, the significance level α was set as 0.05 at two-sided. And the effect size indicates the treatment difference of clinical significance. The primary endpoint is the new-onset neurological symptoms (including pain, numbness and foot drop) of the lower limbs during the perioperative period from LIF surgery until discharge from hospital.

Assuming that the incidence of new-onset lower extremity neurological symptoms before and after the expected improvement [11]was 16% and 5%, respectively, when the two groups with a sample size of 242 subjects are randomly allocated in a 1:1 ratio, a power of 80% to establish superiority of modified LIF sequence over conventional LIF sequence will be reached. If the drop-out rate is 5% or less, a total of 254 eligible subjects would be required to randomly enroll in this study.

### Statistical analysis

The study includes two analysis populations. Of them, Safety set (SS) is defined as all subjects who have received the treatment of the study-specified operation (regardless of whether they participate in the randomized assignment or not) and will be the primary analysis population for safety data. The subjects in SS will be grouped according to the actual surgical treatment received. On the other hand, we will use full analysis set (FAS) for the baseline and efficacy data; It includes all subjects who are randomized into the study groups and received the study operation scheme. Following the principle of intention to treat, subjects in FAS would be analyzed by their randomly assigned group, regardless of the actual operation received.

The primary endpoint in this study is new-onset lower extremity neurological symptoms. The Cochran mantel Haenszel (CMH) method stratified by random stratification factors as appropriate will be used to test the statistical hypothesis. The incidence and 95% confidence interval (CI, Clopper-Pearson method) of will be estimated by treatment group. The CI of incidence difference between the two groups will be obtained by Newcombe method [21]. Similar statistical analysis will further be carried out for individual events of new-onset lower limb neurological symptoms, or Fisher exact method as appropriate will be used for comparison between groups. Additionally, similar analysis for the relief of old lower extremity neurological symptoms will be conducted for pooled and individual events, namely pain, numbness and foot drop during the perioperative period.

For VAS pain score, we will use analysis of covariance (ANCOVA) to compare the between-group changes of the observed values from baseline values after treatment. The random stratification factors as appropriate and the treatment group will be considered as fixed factors with the baseline value as covariate. The least squares mean (LSM), the difference from the control group and its 95% CI will be also provided. Similarly, 3-month recovery rate of lumbar JOA will be analyzed with the use of ANCOVA method.

In addition, we will use a repeated measures mixed effects model (MMRM) as supportive analysis as appropriate. MMRM analysis has treatment group, time and time treatment group interaction as fixed effects, baseline value as covariate, and subjects as random effects. When missing data occur, the last observation carry forward method (LOCF) will be applied to primary analysis and no data imputation as sensitivity analysis. If data distribution limits the use of ANCOVA, a rank-based analysis will be utilized. The LSM, the difference from the control group and its 95% CI will be also provided.

In this study, we will use a *p*-value of 0.05 or less at two-sided to indicate significance for any statistical tests with the use of R, V.4.0.4 [22] and SAS^®^ software, V.9.4 (SAS Institute, North Carolina, USA).

### Ethics and dissemination

#### Ethical considerations

The independent ethics committee (IEC) of Shanghai Changzheng Hospital approved the study protocol (version 1.0, issue date: 2021-05-31) for all three participating centers (Approval No.2021SL030). The IEC agreed that this study will not raise patients’ risk or cause any extra harm to patients. The IEC further agreed that the study is in accordance with the Declaration of Helsinki and that the study will be conducted without ethics problems. All subjects will be required to sign a written informed consent document before their participation in the study.

#### Relevance and dissemination

Our retrospective data (2020) [11] revealed that the modified sequence of LIF surgery can significantly reduce the incidence of immediate post-operative symptoms for patients with single-level lumbar disc herniation via installation of rods prior to insertion of the cage. For the purpose of a higher level of evidence, herein it is expected that this multicentre randomized controlled trial will clearly demonstrate the two types of surgery sequence in terms of immediate post-operative symptoms among diverse LSS patients. It is well known that the adverse experience will significantly compromise the quality of patients’ lives and sometimes even require revision surgery [11, 23, 24].

In the RCT, the conventional LIF surgery sequence will be changed in order: the intervertebral space is first restored and maintained without any distraction or compression force after facetectomy and discectomy; then, differently, the installing and tightening of rods are to be performed prior to the insertion of the cages. By this modified sequence, the inserted cage would be usually smaller than that in the conventional one. In addition, the modified procedure would make it easier to insert an appropriately small cage and even avoid smaller cage to insert.

Given that the immediate postoperative symptoms are available soon after surgery, the study is designed short (approximately three months per subject) in follow-up time. However, it makes sure that we can follow up study patients well and obtain higher quality of data. As regards data analysis, in order to control any possible biases resulting from imbalanced confounders among patients, we will then use stratified randomization technique as is appropriate for this study. This had better help set up any statistical modeling for data analysis. A dedicated data management and analysis team is in place for this study.

In summary, the modified LIF surgery sequence with a rod-prior-to-cage order might be considered more reasonable in treating patients with lumbar spinal stenosis. The study findings will be shared with participating hospitals, and the academic community to promote the clinical management of immediate post-operative symptoms after LIF surgery.

### Trial status

The study is not yet recruiting as of the date of submission.

## Abbreviations

JOA: Japanese Orthopaedic Association
LIF: Lumbar interbody fusion
VAS: Visual analogue scale
